# Comparing patient safety culture in primary, secondary and tertiary hospitals in Ghana

**DOI:** 10.4314/gmj.v57i2.9

**Published:** 2023-06

**Authors:** Aaron A. Abuosi, Emmanuel A. Anaba, Priscilla Y.A. Attafuah, Immaculate S. Tenza, Patience A. Abor, Adelaide Setordji, Edward Nketiah-Amponsah

**Affiliations:** 1 Department of Public Administration and Health Services Management, University of Ghana Business School, Legon, Ghana; 2 Department of Population, Family and Reproductive Health, School of Public Health, University of Ghana; 3 Community Health Nursing Department, School of Nursing and Midwifery, University of Ghana; 4 School of Nursing Science, Faculty of Health Science, North-West University, Potchefstroom Campus, South Africa; 5 South African Research Chairs Initiative (SARChI), School of Public Health, Faculty of Health Sciences, University of the Witwatersrand, Johannesburg, South Africa; 6 Department of Economics, University of Ghana, Legon. Ghana

**Keywords:** Levels of care, patient safety culture, adverse event, patient harm, Ghana, hospital

## Abstract

**Objective:**

This study compared patient safety culture among health professionals in tertiary, secondary and primary hospitals.

**Design:**

We conducted a cross-sectional survey among thirteen primary, secondary and tertiary hospitals in Ghana. A structured questionnaire was administered to 1,656 health professionals. Data were analysed using descriptive statistics and One-Way Analysis of Variance (ANOVA).

**Setting:**

This study was conducted in the Greater Accra, Bono and Upper East regions, representing the southern, middle and northern ecological zones, respectively.

**Participants:**

Health professionals

**Main outcome measures:**

The primary outcome was patient safety culture.

**Results:**

Five patient safety culture dimensions were rated moderate positive response, while five were rated high positive response. We found a statistically significant difference in patient safety culture across primary, secondary and tertiary hospitals (*p* < 0.05). For instance, the mean difference between tertiary and secondary hospitals was statistically significant (*p* < 0.05). Additionally, the mean difference between tertiary and primary hospitals was statistically significant (*p* < 0.05). There was also a significant difference in the means between secondary and primary hospitals (*p* < 0.05).

**Conclusion:**

This study has demonstrated a variation in patient safety culture across Ghana's tertiary, secondary and primary hospitals. Therefore, healthcare managers and professionals should prioritise patient safety.

**Funding:**

This work was supported by the University of Ghana [UGRF/13/MDG-001/2019-2020]

## Introduction

Patient safety is a key indicator of the quality of care and a major concern of healthcare leaders and patients across the globe. Promoting patient safety is crucial due to the unacceptably high burden of adverse events. Patient harm (e.g. pressure ulcers, infusion and drug reactions, patient fall and nosocomial infections) contributes more to the global mortality burden than cancer, diabetes or road injuries.^1^ Medical error, for instance, is a major cause of death globally.^2^-^4^ Besides, patient harm engenders prolonged hospital stay, which contributes to crowding in health facilities and huge healthcare expenditure; hence poses a threat to healthcare quality. 5 Also, unsafe injection practices may lead to infections (i.e. HIV and hepatitis) and pose a hazard to patients and healthcare professionals. For instance, 134 million adverse events are reported annually in low- and middle-income countries, resulting in 2.6 million deaths.^6^ Fortunately, a majority of adverse events are avoidable.^7^ Therefore, the World Health Organization (WHO) aims to eliminate avoidable patient harm by 2030.^6^

Adverse events are reported at all levels of care, including primary, secondary and tertiary hospitals. Evidence shows a significant association between hospital characteristics and adverse events.^8^ Teaching (tertiary) hospitals have a relatively longer stay than non-teaching hospitals.^9^

Evidence suggests that promoting a safety culture in health facilities may help reduce the incidence of adverse events. 10 11 Patient safety culture is “the product of individual and group values, attitudes, perceptions, competencies, and patterns of behaviour that determine the commitment to, and the style and proficiency of, an organization's health and safety management”.^12^-^14^ Patient safety culture should promote a ‘just culture’ through open communication, good information flow, and management commitment.^15^ Additionally, it must support a non-punitive response to error and adverse event reporting.^15^ ^16^ Evidence shows that promoting patient safety culture in health facilities can potentially reduce adverse events and negative patient outcomes.^17^

In Ghana, patient harm is a major concern for patients and health professionals across different levels of care. Existing studies have reported several adverse events in Ghanaian health facilities, including hospital-acquired infections, patient falls, infection abscesses, surgical wound infections, and deaths. 18 19 Patient safety has received the attention of healthcare leaders in Ghana. For instance, Ghana has signed onto WHO's agenda of reducing patient harm by 15% within five years. 20 Ghana is among the three African countries selected to model the African initiative that aims at reducing patient harm by 25% within two years. 21 In addition, Goal 3.1 of the Ghana National Healthcare Quality Strategy (2017-2021) sought to sustain patient safety at all levels of healthcare delivery.^20^

However, patient safety culture has received limited research attention in Ghana. Prior studies focused on specific geographical areas and hospital ownership types. For instance, patient safety culture was perceived to be low in private, mission and public hospitals in the Upper East region.^18^ Another study revealed that patient safety was influenced by teamwork, management support and organizational learning.^22^ These studies set the pace for further studies. The findings could not be generalized to the whole country since the participants were selected in only one out of sixteen regions in Ghana.

Primary hospitals provide primary care and constitute the first point of contact in healthcare delivery. Secondary hospitals focus on referral cases from the primary level, while tertiary hospitals focus on complex medical cases, medical education and research.

The various types of hospitals differ in infrastructure, personnel and funding. However, none of the existing studies in Ghana has investigated patient safety culture across primary, secondary and tertiary hospitals. This study aimed to assess patient safety culture across the three levels of care. Findings from this study can help identify gaps in patient safety culture and inform patient safety policies and programming in the country.

## Methods

### Study design

This study was a cross-sectional survey among health professionals in Ghanaian hospitals.

### Setting

Three administrative regions, including the Greater Accra, Bono and Upper East, were selected to represent the southern, middle and northern ecological zones, respectively. The Greater Accra region has a population of about 5.4 million, the Bono region has a population of 1.2 million, and the Upper East region has a population of 1.3 million.^23^ The Greater Accra region has the highest proportion of health professionals, while the Upper East region has the lowest. Due to resource constraints, we randomly selected Greater Accra, Bono, and Upper East regions to represent the Southern, Middle, and Northern zones. We did not have enough resources to collect data in all sixteen regions of Ghana.

### Participants

Within each selected region, three primary hospitals were purposively selected. The fourth hospital was the regional hospital which is a secondary hospital. One of the five tertiary hospitals in Ghana was purposively selected for this study. The selection of the tertiary hospital was based on the availability of specialised services like oncology and reconstructive surgery. In all, we selected 13 hospitals across the three ecological zones in Ghana. Health facilities below the status of a hospital, such as health centres, clinics and Community-based Health Planning and Services (CHPS) compounds, were excluded. The target population for this study was health professionals (clinical and non-clinical staff), including nurses, doctors, pharmacists, laboratory technicians, radiologists, administrative, and support staff.

### Sample size

The sample size was determined using OpenEpi software based on the following formula:


$$n=deff\times \frac{Npq}{\frac{d^{2}}{1.96^{2}}(N-1)+pq},$$

where n = sample size; *deff* = design effect; N= population size; *p*= estimated proportion; *q*= 1-*p; d*= desired absolute precision /absolute level of precision.

Since the total population of health professionals for the respective regions was unknown, a default value of one million, representing the largest possible population and a 50% hypothesized percentage frequency of the outcome variable was used.^24^ Using a 5% confidence limit and a design effect of 1, the sample size determined for each region was 384. To cater for non-response, 10% of the sample size was added to make it 422. The sample size was divided proportionally to each of the four regional hospitals according to the estimated average annual outpatient attendance. In addition, we selected 422 health workers from the teaching hospital. Thus, the total sample size estimated for this study across the three regions was 1,688 health professionals. However, 1,656 health professionals participated in the study, representing a response rate of 98%. Convenience sampling was used to select participants for the survey. This strategy was appropriate for the nature of this study. 18

### Data sources and variables

The Survey on Patient Safety (SOPS) Culture, Hospital Survey questionnaire (version 2.0) was adapted from the Agency for Health Research and Quality for data collection. The questionnaire has 32 items categorized into ten dimensions. The dimensions were communication openness; staffing and work pace; organizational learning—continuous improvement; hospital management support for patient safety; response to error; supervisor support for patient safety; staffing and work pace; communication about errors; reporting patient safety events; handoffs, and information exchange. The responses were a five-point Likert scale (1: strongly disagree/never to 5: strongly agree/ always). The items were both negatively and positively worded. The questionnaire had a reliability score (Cronbach alpha) ranging from 0.65 to 8.9, considered adequate (0.64 to 0.85).^25^ The questionnaire also captured participant characteristics, including primary work area, position, hours of work, work experience, and direct contact with patients. We added respondents' age, sex, educational level, marital status and religion to the adopted questionnaire.

Data were collected by trained research assistants using Computer Assisted Personal Interviewing (CAPI) application. The questionnaire was administered to participants by trained research assistants using a tablet via a face-to-face approach. Research assistants visited the hospitals, and through the assistance of unit heads, staff who were available and consented were interviewed at a convenient location. The questionnaire was administered in the English language.

Averagely, 45 minutes were spent administering a questionnaire. COVID-19 protocols such as physical distancing and wearing a nose mask were observed.

### Data analysis

Data were analysed using the Statistical Package for Social Sciences (SPSS) (Version 24.0). At the univariate level, descriptive statistics, including frequencies, percentages, graphs and tables, were used to analyse respondents' background data and patient safety culture. One-Way Analysis of Variance was used to test for mean differences between the levels of care and the respective dimensions of patient safety culture. Negatively worded items were reverse-coded before the analysis. All analyses were conducted at a 95% confidence level.

### Ethics and other permissions

This study was approved by the Ghana Health Service Ethics Review Committee (GHS-ERC: 007/04/21) and the Ethics Committee for the Humanities, University of Ghana (ECH 109/20-21). In addition, introduction letters were presented to the heads of the hospitals to elicit their cooperation. Informed consent was obtained from all the participants.

## Results

### Descriptive statistics

It was found that the majority of the participants were females (55%), married (65%) and had tertiary education (89%). About half (49%) of the participants were between the ages of 31 to 40 years, and 14% were Muslims. Regarding the primary work area, 41% of the participants worked in medical/surgical units, while seven in ten participants were nurses. More than half (51%) of the participants had one to five years of work experience in their current hospital, while 55% had one to five years of work experience in their current unit/primary work area. Forty-five per cent of the participants worked more than 40 hours per week, and the majority (96%) had direct contact with patients. (Table 1)

**Table 1:** Study participants' characteristics

Characteristic		Tertiary n (%)	Secondary n (%)	Primary n (%)
**Sex**				
**Male**	739(45)	211(29)	165(22)	363(49)
**Female**	912(55)	224(25)	285(31)	403(44)
**Marital status**				
**Married**	1080(65)	300(28)	289(27)	491(45)
**Unmarried/cohabitating**	571(35)	135(24)	161(28)	275(48)
**Age (years)**				
**30 or below**	594(36)	111(19)	185(31)	298(50)
**31-40**	807(49)	200(25)	220(27)	387(48)
**41 or above**	238(15)	124(52)	45(19)	69(29)
**Educational level**				
**Junior high**	41(3)	7(17)	11(27)	23(56)
**Secondary/Technical**	84(5)	26(31)	31(37)	27(32)
**Post-secondary/others**	43(3)	8(19)	12(28)	23(53)
**Tertiary/University**	1471(89)	394(27)	396(27)	681(46)
**Religion**				
**Catholic**	414(25)	91(22)	128(31)	195(47)
**Protestant**	310(19)	86(28)	90(29)	134(43)
**Pentecostal**	647(39)	185(29)	168(26)	294(45)
**Muslim**	238(14)	65(27)	54(23)	119(50)
**Others**	42(3)	8(19)	10(24)	24(57)
**Primary unit**				
**Medical/surgical**	678(41)	289(43)	149(22)	240(35)
**Emergency care**	108(7)	21(19)	20(19)	67(62)
**Labor/delivery**	292(18)	69(24)	84(29)	139(48)
**Pediatrics**	141(9)	47(33)	42(30)	52(37)
**Specialist service**	184(11)	8(4)	77(42)	99(54)
**Diagnostic**	124(7)	1(1)	48(39)	75(60)
**Administrative/support**	124(7)	0(0)	30(24)	94(76)
**Position/role**				
**Nursing**	1197(72)	341(28)	325(27)	531(44)
**Medical**	175(11)	74(42)	48(27)	53(30)
**Other clinical position**	141(9)	4(3)	42(30)	95(67)
**Managerial/support**	138(8)	16(12)	35(25)	87(63)
**Work experience in current hospital (years)**				
**< 1**	286(17)	43(15)	97(34)	146(51)
**1-5**	841(51)	196(23)	231(27)	414(49)
**>5**	524(32)	196(37)	122(23)	206(39)
**Work experience in the current unit (years)**				
**< 1**	452(27)	90(20)	141(31)	221(49)
**1-5**	907(55)	192(21)	268(30)	447(49)
**>5**	292(18)	153(52)	41(14)	98(34)
**Work hours per week 30-40**	910(55)	232(25)	264(29)	414(45)
**> 40**	741(45)	203(27)	186(25)	352(48)
**Direct contact with patients**				
**Yes**	1581(96)	432(27)	429(27)	720(46)
**No**	70(4)	3(4)	21(30)	46(66)

### Health professionals' perceptions of patient safety culture

Of the ten patient safety culture dimensions, teamwork (88.7%) scored the highest average positive response rate, while reporting of patient safety events scored the lowest positive response (54.6%). Five dimensions were rated moderate (50% to 69.9%). These include staffing and work pace (60.4%); error response (59.8%); communication openness (65.7%); reporting of patient safety events (54.6%); and hospital management support for patient safety (66.5%).

On the other hand, five dimensions were rated high (> 70%). These include teamwork (88.7%); organizational learning-continuous improvement (81%); supervisor support for patient safety; communication about errors; and handoffs and information exchange (81.6%). (Table 2)

**Table 2:** Health professionals' perceptions of patient safety culture

Dimensions of patient safety culture	% of average positive response
**Teamwork**	88.7
**Supervisors support patient safety**	83.8
**Handoffs and information exchange**	81.6
**Organizational learning**	81.0
**Communication about error**	72.5
**Hospital management support**	66.5
**Communication openness**	65.7
**Staffing and work pace**	60.4
**Response to error**	59.8
**Reporting patient safety events**	54.6

***(Interpretation of positive response: below 50% = low; 50-69.9% = moderate; 70% or above = high)***	

### One-way Analysis of Variance for patient safety culture dimensions across levels of care

We compared patient safety culture dimensions across primary, secondary and tertiary hospitals. Generally, patient safety culture was relatively poor in the tertiary hospital. Regarding teamwork, there was a statistically significant difference across primary, secondary and tertiary hospitals (*P* < 0.05). Similarly, we found a significant difference in some dimensions. For example, response to error, staffing and work pace; organizational learning-continuous improvement; and supervisor support for patient safety (*P* < 0.05). A post-hoc analysis using the Bonferroni test showed that the mean difference between the tertiary and secondary hospitals was statistically significant (*P* < 0.05). Also, the mean difference between the tertiary and primary hospitals was statistically significant. Moreover, we found a significant difference in the means of the secondary and primary hospitals. The effect size was estimated using the *Cohen F* technique^26^, which revealed that the effect size ranged from small to large. For instance, organizational learning and continuous improvement had the largest effect size of 0.43, while teamwork had a medium effect size of 0.30. 26 A large effect size implies that the finding has practical significance. However, hospital management support for patient safety, handoffs and information exchange did not differ significantly across primary, secondary and tertiary hospitals (*p* > 0.05). (Table 3)

**Table 3:** One-way ANOVA comparing patient safety culture dimensions across levels of care

Characteristic					Effect-size	Post-hoc analysis for mean difference (Bonferroni)		

Dimensions	Tertiary (A) m(sd)	Secondary (B) m(sd)	Primary (C) m(sd)	*P*-value	*Cohen F*	A-B	A- C	B-C
**Teamwork**	3.92(0.6)	4.24(0.5)	4.30(0.6)	< 0.001	0.30	-0.32*	-0.38*	-0.05
**Staffing and work pace**	3.21(0.6)	3.61(0.6)	3.40(0.6)	< 0.001	0.22	-0.39*	-0.19*	0.20*
**Organizational learning**	3.47(0.6)	4.09(0.5)	4.04(0.6)	0.001	0.43	-0.61*	-0.56*	0.52
**Response to error**	3.26(0.7)	3.57(0.7)	3.38(0.8)	0.006	0.14	-0.31*	-0.12*	0.19*
**Supervisor support**	3.72(0.6)	4.00(0.6)	4.03(0.6)	< 0.001	0.20	-0.27*	-0.30*	-0.02
**Communication about error**	3.91(0.6)	4.06(0.6)	3.92(0.7)	< 0.001	0.10	-0.14*	-0.001	0.14*
**Communication openness**	3.83(0.6)	3.94(0.6)	3.77(0.7)	< 0.001	0.10	-0.10*	0.05	0.16*
**Reporting events**	3.60(0.9)	3.73(0.9)	3.34(0.9)	< 0.001	0.17	-0.12	0.26*	0.39*
**Management support**	3.45(0.7)	3.54(0.7)	3.53(0.8)	0.178				
**Handoffs & exchange**	3.86(0.5)	3.93(0.7)	3.86(0.7)	0.21				

* Note: P-Value < 0.05; Effect Size (*Cohen F*): 0.10 Small, 0.25- Medium, 0.40- Large

## Discussion

The findings show that patient safety culture differed significantly across primary, secondary and tertiary hospitals. Eight of the ten patient safety culture dimensions showed a statistically significant difference across tertiary, secondary and primary hospitals. Generally, patient safety culture was rated low in the tertiary hospital compared to primary and secondary hospitals. For instance, organizational learning was rated low in the tertiary hospital compared to the secondary and primary hospitals. The effect size was high, suggesting a strong practical significance. In addition, teamwork was rated low in the tertiary hospital compared to the secondary and primary hospitals. The effect size was considered medium, which suggests a moderate practical significance.

A previous study corroborated that patient safety culture was poor in a tertiary hospital in Ghana.^19^ Similar to the findings of our study, the authors found that teamwork had a high positive response, while adverse event reporting had low positive response ratings.^19^ These findings are understandable, considering the health system challenges in Ghana. Currently, only five tertiary hospitals in Ghana are expected to serve a population of about thirty-one million. These few tertiary hospitals receive referral cases from several primary and secondary hospitals nationwide. In addition, some patients abuse the gatekeeper system by using tertiary hospitals as their first point of contact instead of visiting primary health facilities. 27 Hence, tertiary hospitals may be overwhelmed with medical conditions and many patients amid resource constraints, affecting their ability to report adverse events. Similarly, a previous study reported that high patient attendance was a major factor in the failure to report medical errors.^28^ Despite the preceding possible justification, the low reporting may be attributed to healthcare organisations' blame (punitive) culture. The literature is conclusive that blaming culture in healthcare is a major reason for the under reporting of medical errors.^29^-^31^

The low reporting of adverse events across primary, secondary and tertiary hospitals in Ghana is a major setback that requires the attention of stakeholders. The tertiary hospital is the highest level of care in Ghana, where complex medical cases are managed. Hence standards of care are expected to be better than the primary and secondary hospitals. Therefore, it is a matter of concern that reporting of adverse events, which is an important indicator of a high standard of care, is low in tertiary hospitals compared with primary and secondary hospitals. Stakeholders, including the Ministry of Health and Ghana Health Service leaders, can leverage these findings to inform patient safety policies and strategies. It is recommended that healthcare leaders should focus on promoting a just culture by adopting a non-punitive response to errors and anonymous error reporting strategies to help improve medical error reporting in Ghanaian hospitals.

The findings of this study provide useful information that can inform patient safety programming, policy and research. This study provides relevant information for improving patient safety culture among health professionals. In addition, we used a standardized questionnaire that has been adopted globally. Hence these findings can be juxtaposed with findings across the globe. Notwithstanding, this study is subject to social desirability biases. Participants may rate themselves positively, which might have accounted for the high rating of patient safety culture. Another limitation of this study is the convenient selection of participants; hence the findings should be interpreted with this limitation in mind.

## Conclusion

The study demonstrated that patient safety culture is a major concern across Ghana's primary, secondary and tertiary hospitals. However, patient safety culture in primary hospitals differs from the secondary and tertiary hospitals. The findings also underscored areas of patient safety culture that require improvement.
